# Mastering the learning curve of endoscopic mitral valve surgery

**DOI:** 10.3389/fcvm.2023.1162330

**Published:** 2023-06-22

**Authors:** Luca Aerts, Peyman Sardari Nia

**Affiliations:** Department of Cardiothoracic Surgery, Maastricht University Medical Center, Maastricht, Netherlands

**Keywords:** mitral valve surgery, minimally invasive surgery, learning curve, surgical simulation, surgical training

## Abstract

Endoscopic mitral valve surgery is a challenging procedure. Surgical volume is mandatory to achieve sufficient proficiency and superior results. To this date the learning curve has proven to be challenging. Offering high-fidelity simulation based training for both residents as experienced surgeons can help in establishing and enlarging surgical competences in shorter time without intraoperative trial and error.

## Introduction

Endoscopic mitral valve surgery was first introduced in 1966 by Alain Carpentier (1). Over the years this challenging procedure improved extensively by the development and refinement of patient selection, (pre)-operative planning, surgical techniques and simulation based training (2, 3). Minimally invasive approaches offer the possibility of superior surgical and post-operative outcomes by enhanced dexterity (4). However, surgical volume is mandatory to achieve sufficient proficiency and superior results (5, 6). To this date the learning curve has proven to be challenging. Literature suggest in order to perform minimally invasive mitral valve surgery (MIMVS), a surgeon will need to complete 75 to 125 operations to overcome the associated learning curve (6).

The past decades, MIMVS is increasingly accepted by cardiothoracic surgeons and is nowadays even the golden standard in few highly-experienced centers (7). As a result, patient demand is increasing, which may lead to more surgeons adopting the MIMVS technique (8).

To facilitate in this growing interest in MIMVS, an enhanced learning platform was developed. Offering high-fidelity simulation based training for both residents as experienced surgeons. This framework can help in establishing and enlarging surgical competences in shorter time without intraoperative trial and error (3).

## The learning curve

**Figure 1:**
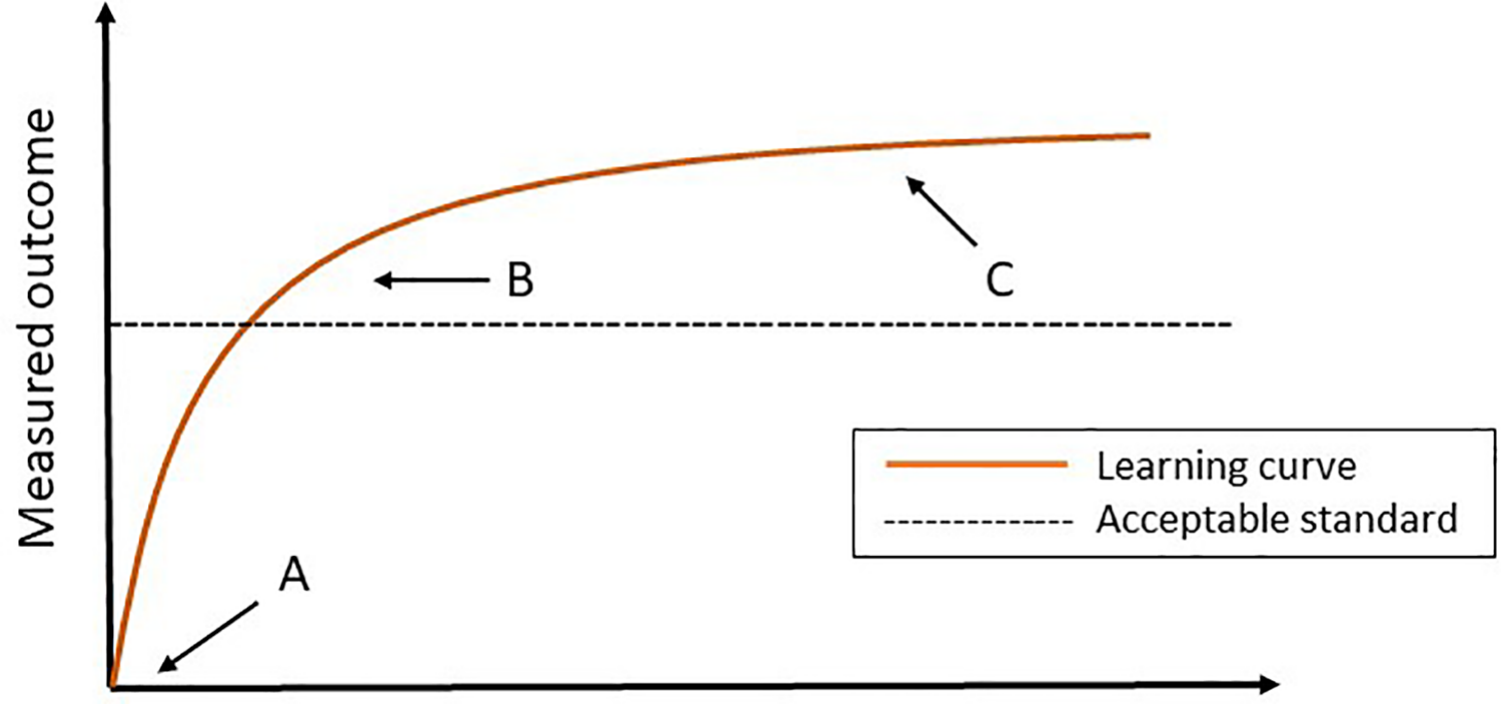
The learning curve. (**A**) The starting point. (**B**) The slope (the surgeon can perform the procedure independently and competently). (**C**) Plateau phase.

Mastering a new surgical procedure, especially technical demanding procedures such as minimally invasive surgery, requires surgical volume. Performances tend to improve with experience and when plotting this performance graphically against experience, a learning curve arises. This curve is roughly characterized by three phases; the starting point, the slope, where a surgeon in training improves, and the plateau-phase (Figure 1) (9).

The course of this curve is determined by multiple factors, such as the surgical workload, the technical equipment at your disposal, a competent learning program and patient specific factors, such as anatomy. In addition, the innate ability and other non-technical skills of the individual resident or surgeon contribute to the learning curve and should not be overlooked (10, 11). Summarily, to overcome this learning curve, one should be aware of the significance of improvements in a multifactorial performance environment.

Recent literature shows higher surgical volume is significantly associated with improved outcomes of repair rate (OR = 1.25–5.5) and mortality (OR = 0.46–0.84 and OR = 1.5–2.27 depending on the reference group) in mitral valve surgery. A mean threshold of minimally 30 mitral valve procedures per year was calculated (5). For MIMVS data is limited.

Holzhey et all (2013) elaborated on this and analyzed 5,287 patients in their center. Surgeons who performed less than 20 MIMVS operations were excluded, so that eventually 3,895 operations performed by 17 surgeons were analyzed. A clear tendency towards better results was observed, when a surgeon's experience in MIMVS procedures increased. They indicated MIMVS requires 75 to 125 procedures per year to overcome its associated learning curve. Furthermore, they stated it's fundamental for a surgeon to perform at least 1 MIMVS procedure per week to maintain proficiency. However, as the authors did not perform all procedures endoscopic, the results cannot be translated one-to-one into practice (6).

## Structural training for cardiothoracic surgery

For decades, training on real patients in the operating room was one of the most powerful approaches used in medical training to master the learning curve of a procedure. But, against the backdrop of working hours and rising developments of novel techniques, there is a greater public scrutiny and accountability about the skill acquisition process in surgery (12). This has moved the medical community in the 20th century to develop a different learning platform, consisting of VR modalities and simulation based training, to provide structural training outside the operative theatre.

Structural training for cardiothoracic surgery and MIMVS in general is lacking uniformity across Europe. Due to the different technical strategies in terms of access, vision, perfusion techniques and conditioning, there is no scientific-based consensus regarding standardization in MIMVS. Subsequently, there is no guidance by an outcome based syllabus for structural training, making it hard to master the challenging learning curve.

**Figure 2:**
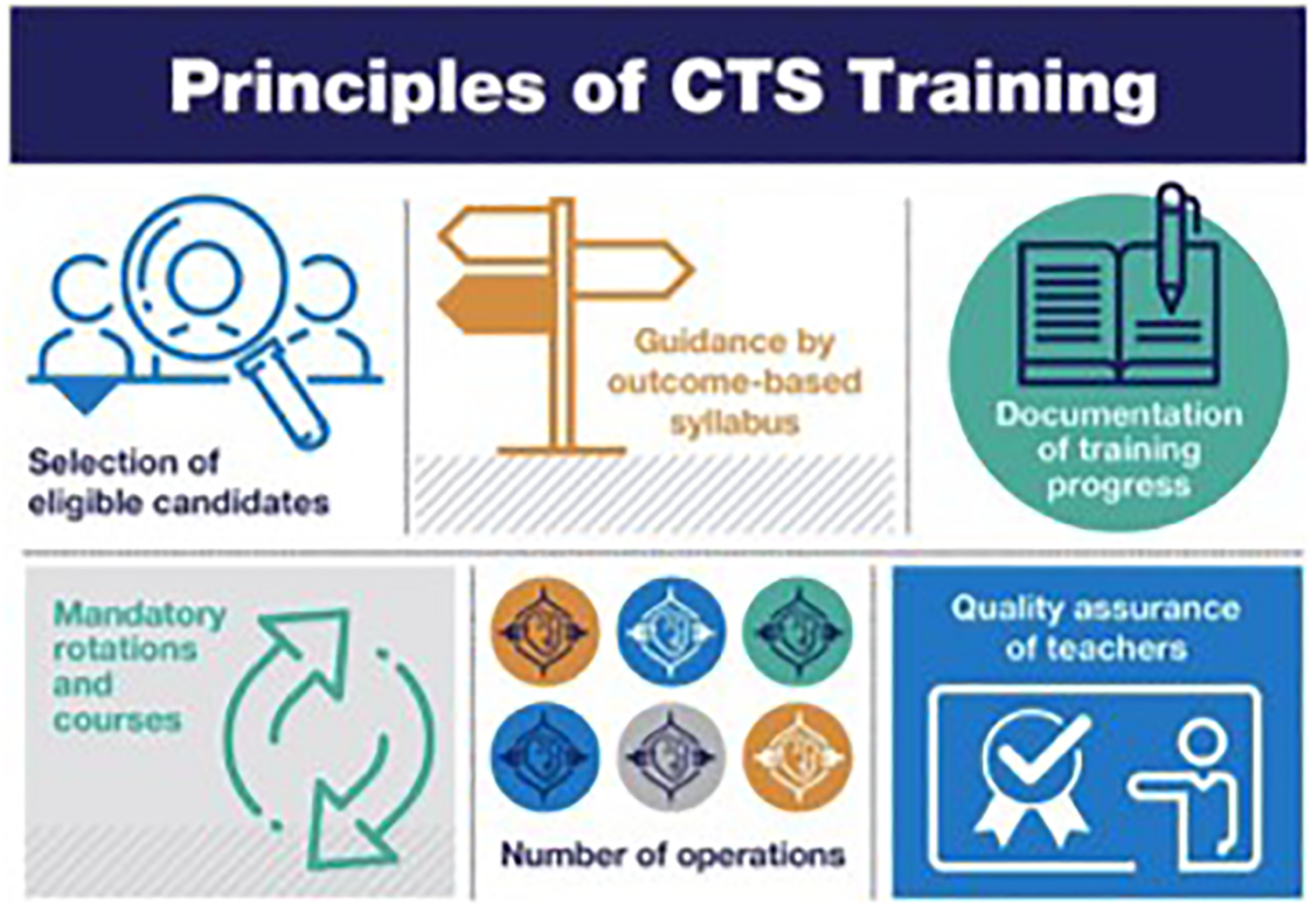
The principles of cardiothoracic surgery. Reprinted from Interactive CardioVascular and Thoracic Surgery, IVAC 213, Zientara et al. Basic principles of cardiothoracic surgery training: a position paper by the European Association for Cardiothoracic Surgery Residents Committee, 2022 (14).

Various efforts have been made to outline the fundamentals of an ideal training program, bearing the paradigm shift from an apprenticeship to a competency-based model in mind (13). Zientara et al. (2019) defined the key structural, administrative and executive principles of cardiothoracic surgery training as shown in Figure 2 (14).

All the principles elucidated for cardiothoracic surgery can also be applied to MIMVS. Patient selection is critical in MIMVS, especially in the early stages of skill development. The most appropriate patient to start with is a vital young patient with minimal comorbidities and anatomically suitable for the minimal invasive approach. Patients with high BMI, significant comorbidities and impaired cardiac function should be carefully considered, particularly early in the training program (15).

Additionally, another basic principle for medical education is to provide mentorship. A skilled surgical coach could provide formative feedback and recognize the areas of weakness requiring further practice or remediation (16).

At present, we are trained in a culture that emphasizes and rewards individual achievements. However, the importance of teamwork in medical practice is inevitable for improving care and patient safety. Recent literature shows that patients who are treated for mitral valve disease on a dedicated heart team decision have significantly higher survival independent of baseline characteristics mitral valve pathology and allocated treatment (17). With all these assumptions, the concept of simulation based training was further developed and fine-tuned.

## Simulation based training

Simulation based training is a technique for practice and learning to amplify real-time experiences in fully interactive manner (18). This type of educational endeavor has been well described for laparoscopic surgery and proved to be effective in reducing the learning curve of *in vivo* procedures (19). In order to successfully incorporate the simulator into a training curricula, there is a need for objective assessment. The tool should provide formative and objective feedback.

**Figure 3:**
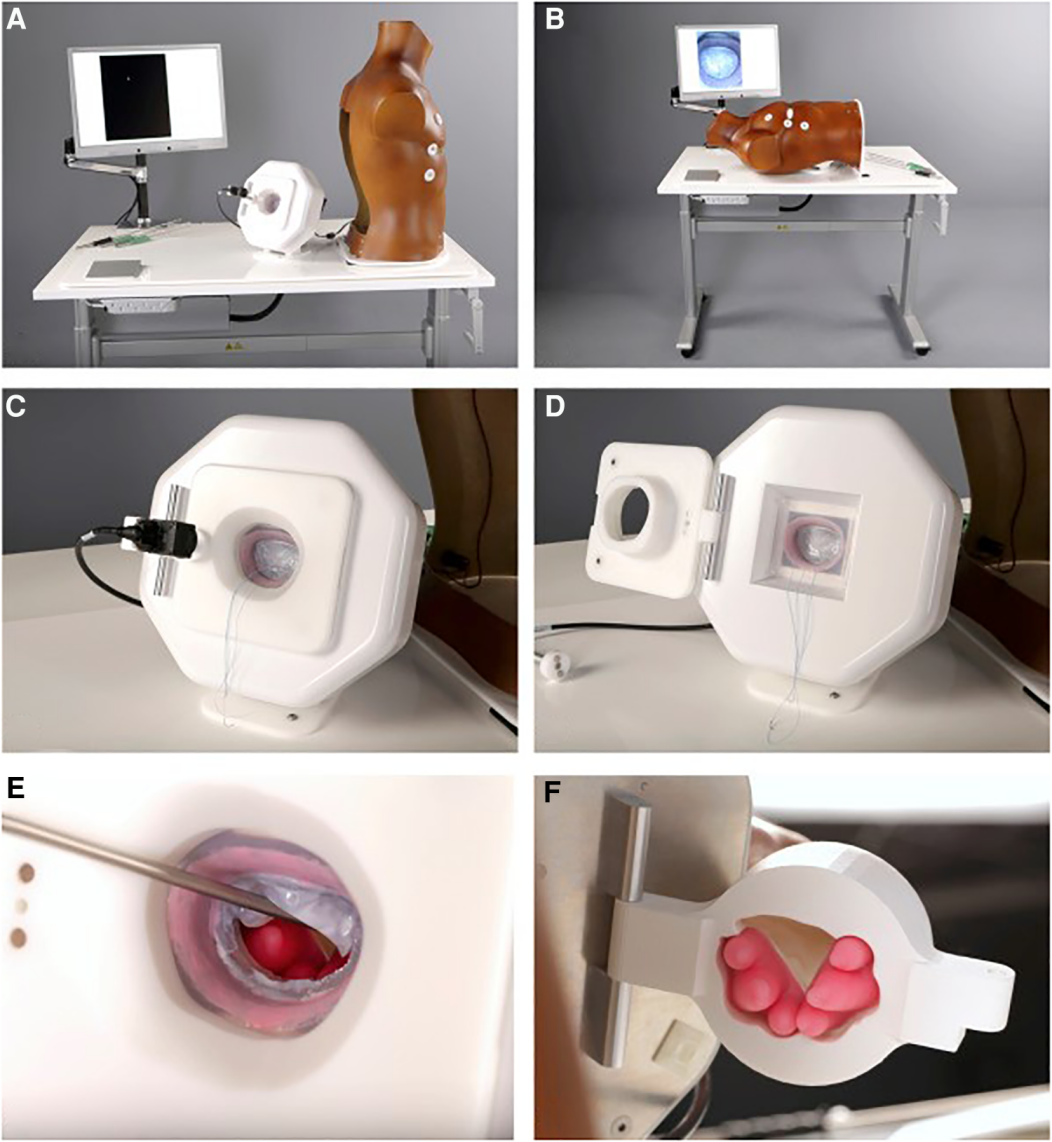
Final version of the simulator. (**A,B**), View of the assembled simulator. (**C,D**), Feedback system. (**E,F**), Magnetic, silicone papillary muscles mounted in the 3-dimensional–printed ventricle. *Reprinted from The Journal of Thoracic and Cardiovascular Surgery, Volume 157, Nia, P. S., Daemen, J. H., & Maessen, J. G, Development of a high-fidelity minimally invasive mitral valve surgery simulator, 1567-1574, 2019, with permission from Elsevier* (3)*.*

In mitral valve surgery, various simulators and training models have been developed (20–23). However, all the above-mentioned simulators are defined as low-fidelity and evidence regarding its usability and efficacy is restrictive. Fidelity in simulation can be defined as a multi-dimensional concept. Fidelity is related to the degree of realism, which is determined by its equipment, scenario and setting, and the degree of exactness achieved. Therefore, a low-fidelity simulator can be interpreted as a black box, lacking feedback. For this reason, we developed a high-fidelity simulator as educational tool for MIMVS in 2012, providing objective, reproducible and metric-based feedback (Figure 3) (3).

### High fidelity simulator in mitral valve surgery

**Figure 4:**
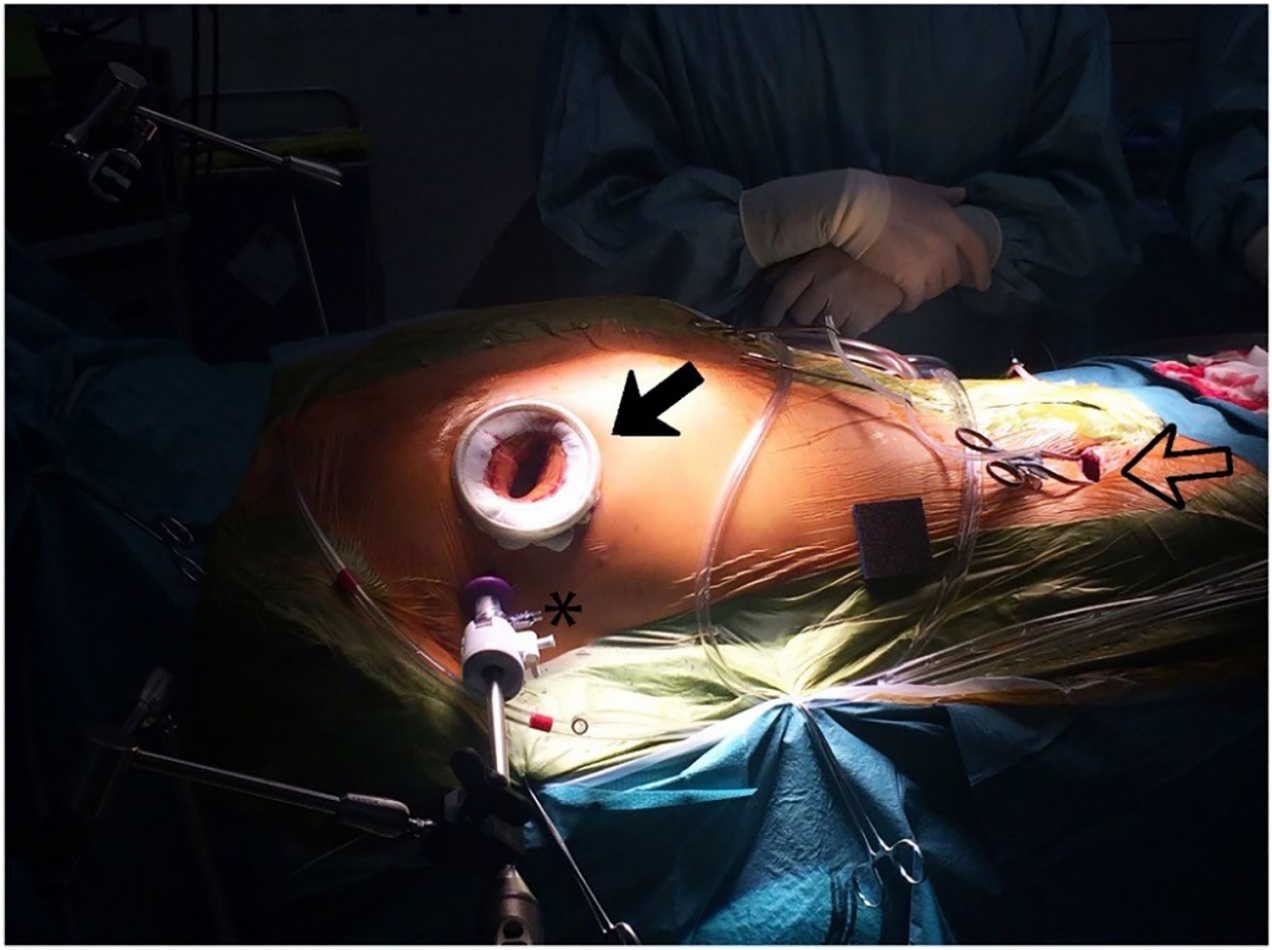
Black outlined arrow: right sided minithoracotomy with soft tissue retractor; asterisk: 3-dimensional (3D) endoscope placed through a trocar in the same intercostal space; black arrow: peripheral cannulation in the right groin, *with permission of journal of visualized surgery* (24).

The high-fidelity simulator consists of a thoracic torso with a window at the fourth intercostal space mimicking a real-time port access setting. This unique angle of view and limited access for the long-shafted instruments is part of the training in “chopstick” surgery, such as MIMVS (Figure 4).

When lifting the torso, there is a model which resembles the mitral valvular apparatus with a slightly dilated atrium. The mitral valve is casted in a deformable silicone model using 3D printing, providing a true-to-nature suture experience (Figure 3E). The papillary muscles, situated behind the valvular apparatus, are mounted using magnets to create awareness of its vulnerability in simulation based training (Figure 3F). We have shown the feasibility of these silicone models and described its's usability for training purposes and preoperative planning of a complex MIMVS procedure (25).

**Figure 5:**
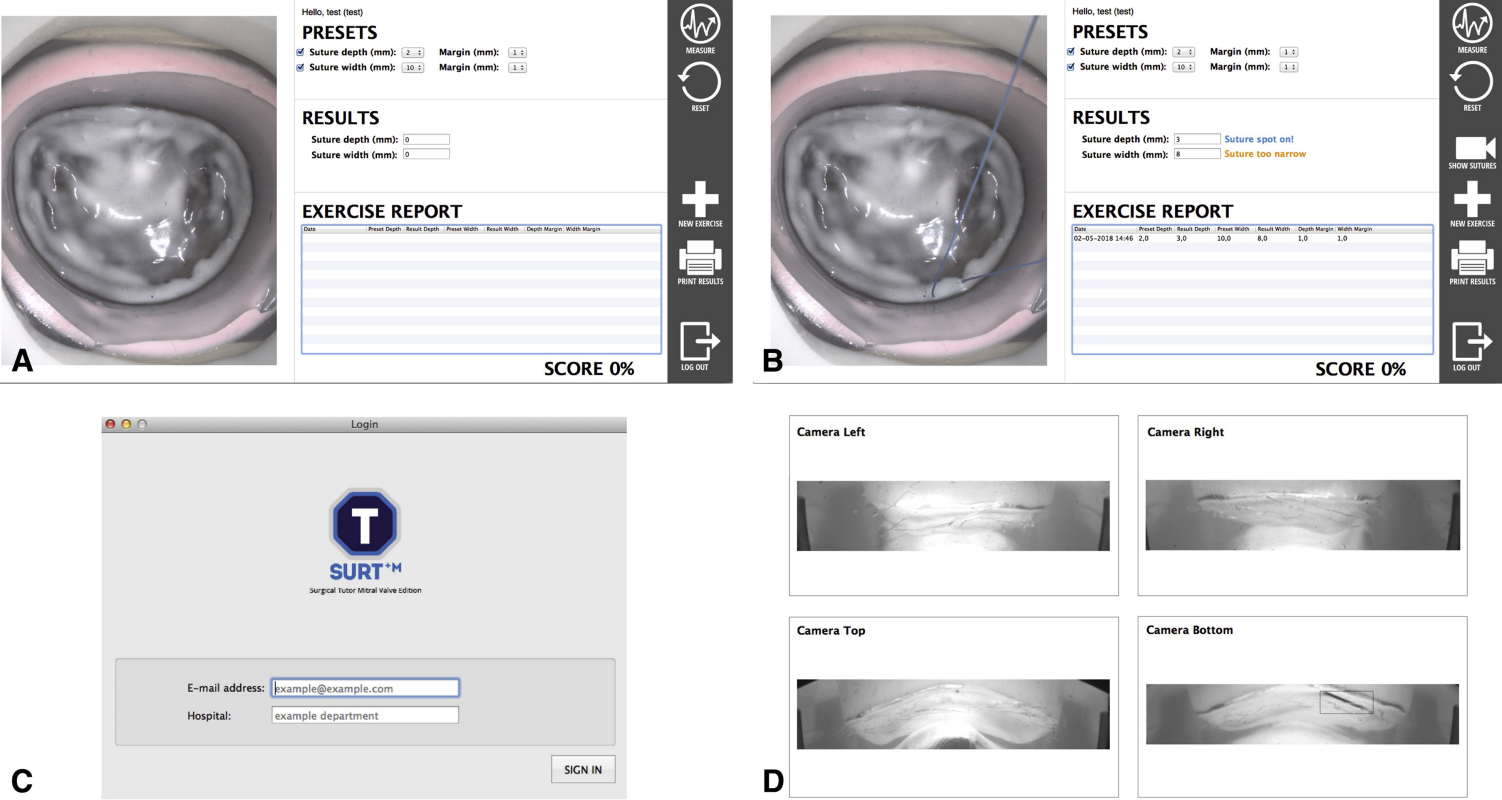
User interfaces of the feedback system. (**A,B**). Graphical user interface that provides on-screen feedback, the endoscopic view and allows the user to define the margin of error. (**C**). Log-in screen. (**D**). Internal camera from which width and depth are measured, *with permission of The Journal of thoracic and cardiovascular surgery* (3).

The great advantage of this simulator is the feedback system incorporated in this model, as highlighted before feedback is the cornerstone of education (26). Four cutouts were made in the aluminum housing of the silicone model to fit four high-resolution cameras. When placing a suture in the mitral valve model, an edge detectable algorithm calculates the suture width and depth (Figure 5). These specific feedback performance measures are clearly identified characteristics that are defined by experienced surgeons. This way, the feedback is objective, consistent and reproducible and can serve as an effective tool to develop skill acquisition (27).

In order to validate the simulator, 99 independent surgeons evaluated the model by questionnaire statements, scored on a 1-to 5-point Likert scale. All the correspondents agreed that the MIMVS simulator is a realistic and useful tool for training purposes in MIMVS (3). The next step was to test if the simulator actually improves trainee performance. For this purpose we designed an air-pilot training concept (Endoscopic Mitral Valve Repair Course) (28).

Although results are promising, there are several limiations using the above-mentioned simulator for training purposes in MIMVS. First of all, the model used in simulation based training is not fully representative of the heart. Futhermore, the focus of the simulator is directed toward annular suture placement and sealing the annuloplasty ring, instead of the overall scope of MIMVS. This educational tool does not provide training how to access the valve without complications. In our opinion, a basic level of surgical skills should be obtained before starting the program.Joyce et al. reported on the restricted home practice (3 h during 2 weeks). (15) This in mind, the program should stress the imperative need for a concept of deliberate practice in (residency) training.

## Training course

Prospective, randomized, double-blinded studies have proven that residents in surgery, trained with high fidelity simulators, have significantly less intra-operative inaccuracies (14–29). Therefor, we believe simulation based training can influence the steepness of the learning curve by providing fidelity on performance sensing, assessment and procedural rehearsal (27).

**Figure 6:**
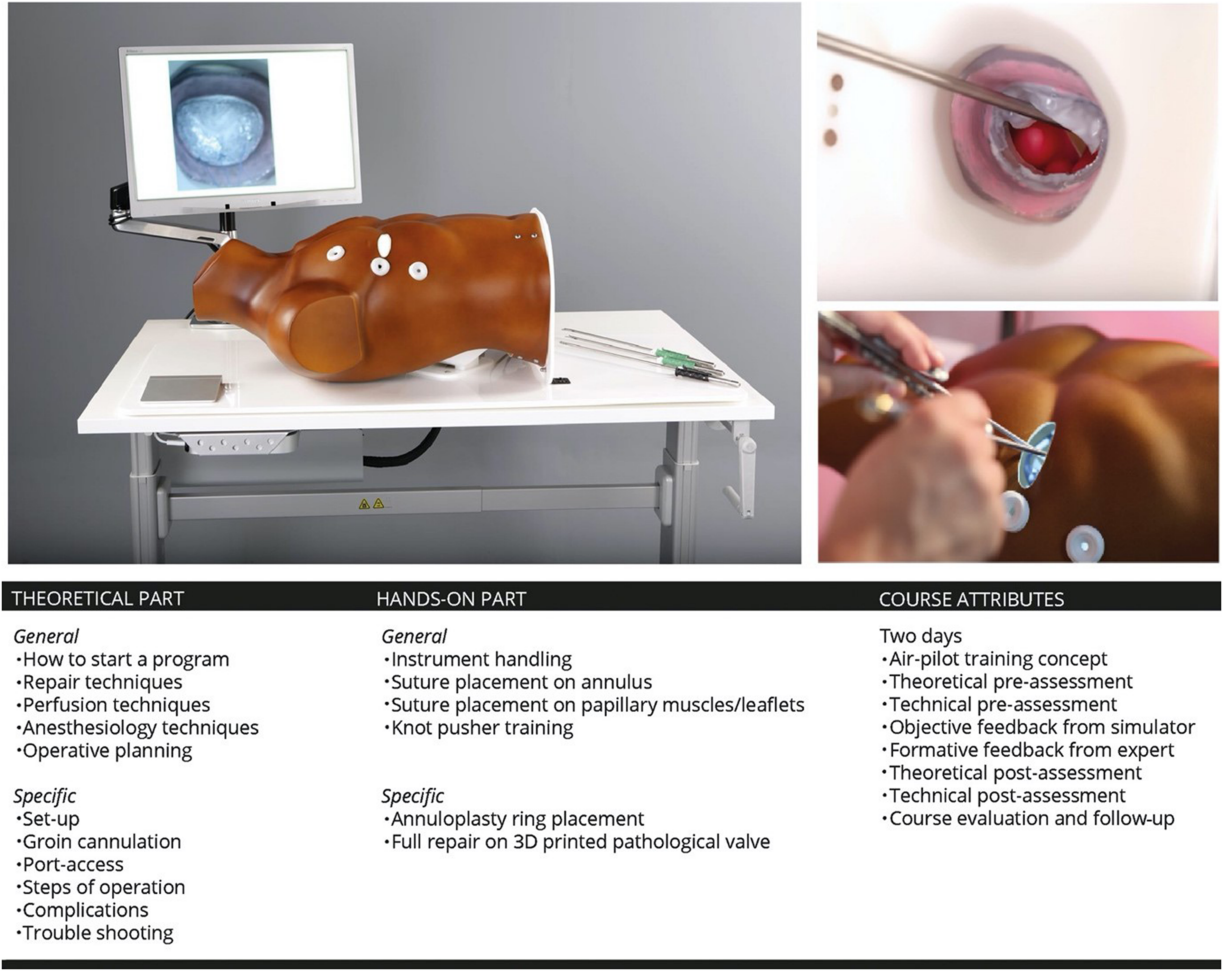
Overview of the philosophy of simulation based training and its inclusion in the endoscopic mitral valve course in our centre. 3D: three-dimensional. *Reprinted from Interactive CardioVascular and Thoracic Surgery, Volume 30, Issue 5, Peyman Sardari Nia, Samuel Heuts, Jean H T Daemen, Jules R Olsthoorn, W Randolph Chitwood, Jr, Jos G Maessen, The EACTS simulation-based training course for endoscopic mitral valve repair: an air-pilot training concept in action, 691-698, 2020, with permission from Elsevier* (28).

We designed an air-pilot training concept (Endoscopic Mitral Valve Repair Course) in collaboration with the European Association for Cardiovascular Surgery (EACTS). The two day course started with a theoretical and technical pre-assessment on the simulator. During the course a MIMVS expert elaborated on the theoretical part of MIMVS and provided together with the simulator formative and metric based feedback on the technical skills of the participants. At the end of the course the same theoretical and technical assessment was evaluated (Figure 6).

We analyzed 102 participants who attended the full course. The analyzed participants consisted of 83 (83.3%) staff surgeons, 12 (11.8%) surgeons who finished residency and 5 (4.9%) residents in training. The participants showed significant higher theoretical knowledge of MIMVS after completion of the course (median score 58% vs. 67%, *P* < 0.001) and their technical skills improved significantly in suture accuracy (43% vs. 99%, *P* < 0.001) and speed (87 s vs. 42 s, *P* < 0.001). This study proved that the high-fidelity simulator is a validated tool to provide in training purposes for MIMVS (28).

In the course evaluation afterwards, 33% of the participants stated they started the MIMVS program successfully. The other 67% of participants did not started the program. Of these 23.7% had no intention of starting the program, in contrast to 76.3% who stated they wanted to start the program in the near future. The following reasons for not starting the program were given: lack of collegial support, lack of case or volume load and lack of facilities (28). In light of these promising findings, we should envision a training program, rather than a single course in MIMVS to master its associated learning curve.

## Future perspectives

Firstly, simulation based training should not be seen as a substitute, but rather an additional tool for structural training in MIMVS. For future, we envision to develop and implement worldwide training programs. To achieve this, simulators should be incorporated in clinical guidelines as a potential training pathway. Simulation based training in MIMVS should be part of national scientific session meetings and international fellow education. In order to provide in this trajectory, we want to increase the accessibility of simulators at the educational institute or at home to continue practicing to master its associated learning curve.

**Figure 7:**
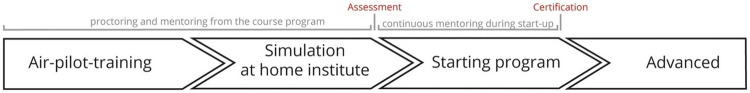
Timeline of the air-pilot training concept programme with inclusion of the training at our course, simulation and proctoring at the participants’ home centre and actual start of a self-managed program. *Reprinted from Interactive CardioVascular and Thoracic Surgery, Volume 30, Issue 5, Peyman Sardari Nia, Samuel Heuts, Jean H T Daemen, Jules R Olsthoorn, W Randolph Chitwood, Jr, Jos G Maessen, The EACTS simulation-based training course for endoscopic mitral valve repair: an air-pilot training concept in action, 691-698, 2020, with permission from Elsevier* (28).

In order to provide a successful concept training program for residents and surgeons interested in MIMVS, one should have first of all inclusion and exclusion criteria. Once the surgeon in training for MIMVS completed the two day MIMVS air-pilot-training course, one should get access to a simulator available for home-use. Subsequently, intensive fellowship will follow to provide the trainee insights regarding patient selection, (pre)-operative planning and surgical technique in real patients. During the training program proctoring and mentorship continues remotely. Finally, objective certification based on the program success should be the icing on the cake (Figure 7).

## References

[1] Carpentier A Loulmet D Carpentier A Le Bret E Haugades B Dassier P Open heart operation under videosurgery and minithoracotomy. First case (mitral valvuloplasty) operated with success. C R Acad Sci III. (1996) 319:219–23. 8761668

[2] Heuts S Olsthoorn JR Maessen JG Nia PS. Planning minimally invasive mitral valve surgery. J Vis Surg. (2018) 4:212–22. 10.21037/jovs.2018.09.07

[3] Nia PS Daemen JH Maessen JG. Development of a high-fidelity minimally invasive mitral valve surgery simulator. J Thorac Cardiovasc Surg. (2019) 157(4):1567–74. 10.1016/j.jtcvs.2018.09.014 30385017

[4] Mohiuddin K Swanson SJ. Maximizing the benefit of minimally invasive surgery. J Surg Oncol. (2013) 108(5):315–9. 10.1002/jso.23398 24037974

[5] Akmaz B van Kuijk SM Nia PS. Association between individual surgeon volume and outcome in mitral valve surgery: a systematic review. J Thorac Dis. (2021) 13(7):4500. 10.21037/jtd-21-578 34422376 PMC8339780

[6] Holzhey DM Seeburger J Misfeld M Borger MA Mohr FW. Learning minimally invasive mitral valve surgery: a cumulative sum sequential probability analysis of 3895 operations from a single high-volume center. Circulation. (2013) 128(5):483–91. 10.1161/CIRCULATIONAHA.112.001402 23804253

[7] Kempfert J Kofler M Falk V Sündermann SH. Minimally invasive endoscopic mitral valve repair-the new gold standard for degenerative mitral valve disease. Eur J Cardiothorac Surg. (2022) 61(3):645–6. 10.1093/ejcts/ezab568 35025989

[8] Dagenais F. Minimally invasive mitral valve surgery: evolution, techniques and outcomes (2008).

[9] Hopper AN Jamison MH Lewis WG. Learning curves in surgical practice. Postgrad Med J. (2007) 83(986):777–9. 10.1136/pgmj.2007.057190 18057179 PMC2750931

[10] Subramonian K Muir G. The “learning curve” in surgery: what is it, how do we measure it and can we influence it? BJU Int. (2004) 93(9):1173–4. 10.1111/j.1464-410X.2004.04891.x 15180598

[11] Moran BJ. Decision-making and technical factors account for the learning curve in complex surgery. J Public Health (Oxf). (2006) 28(4):375–8. 10.1093/pubmed/fdl048 16870993

[12] Connors RC Doty JR Bull DA May HT Fullerton DA Robbins RC. Effect of work-hour restriction on operative experience in cardiothoracic surgical residency training. J Thorac Cardiovasc Surg. (2009) 137(3):710–3. 10.1016/j.jtcvs.2008.11.038 19258094

[13] Loubani M Sadaba JR Myers PO Cartwright N Siepe M Emmert MY A European training system in cardiothoracic surgery: is it time? Eur J Cardiothorac Surg. (2013) 43:352–7. 10.1093/ejcts/ezs208 22518040

[14] Zientara A Hussein N Bond C Jacob KA Naruka V Doerr F Basic principles of cardiothoracic surgery training: a position paper by the European association for cardiothoracic surgery residents committee. Interact Cardiovasc Thorac Surg. (2022) 35(4):ivac213. 10.1093/icvts/ivac213 36018268 PMC9479886

[15] Barac YD Glower DD. Port-Access mitral valve surgery—an evolution of technique. Semin Thorac Cardiovasc Surg. (2020) 32(4):829–37. WB Saunders. 10.1053/j.semtcvs.2019.09.003 31518704

[16] Ericsson KA Krampe RT Tesch-Römer C. The role of deliberate practice in the acquisition of expert performance. Psychol Rev. (1993) 100(3):363. 10.1037/0033-295X.100.3.363

[17] Nia PS Olsthoorn JR Heuts S van Kuijk SMJ Vainer J Streukens S Effect of a dedicated mitral heart team compared to a general heart team on survival: a retrospective, comparative, non-randomized interventional cohort study based on prospectively registered data. Eur J Cardiothorac Surg. (2021) 60(2):263–73. 10.1093/ejcts/ezab065 33783480

[18] Gaba D. The future of simulation in health care. Qual Saf Health Care. (2004) 13:2–10. 10.1136/qshc.2004.009878 15465951 PMC1765792

[19] Aggarwal R Ward J Balasundaram I Sains P Athanasiou T Darzi A. Proving the effectiveness of virtual reality simulation for training in laparoscopic surgery. Ann Surg. (2007) 246(5):771–9. 10.1097/SLA.0b013e3180f61b09 17968168

[20] Joyce DL Dhillon TS Caffarelli AD Joyce DD Tsirigotis DN Burdon TA Simulation and skills training in mitral valve surgery. J Thorac Cardiovasc Surg. (2011) 141(1):107–12. 10.1016/j.jtcvs.2010.08.059 21074189

[21] Verberkmoes NJ. Verberkmoes-Broeders E.M.P.C. A novel low-fidelity simulator for both mitral valve and tricuspid valve surgery: the surgical skills trainer for classic open and minimally invasive techniques. Interact Cardiovasc Thorac Surg. (2013) 16:97–101. 10.1093/icvts/ivs451 23125307 PMC3548533

[22] Hossien A. Low-fidelity simulation of mitral valve surgery: simple and effective trainer. J Surg Educ. (2015) 72:904–9. 10.1016/j.jsurg.2015.04.010 26116402

[23] Greenhouse DG Grossi EA Dellis S Park J Yaffee DW DeAnda A Jr Assessment of a mitral valve replacement skills trainer: a simplified, low-cost approach. J Thorac Cardiovasc Surg. (2013) 145(1):54–9. 10.1016/j.jtcvs.2012.09.074 23111016

[24] Olsthoorn JR Heuts S Attaran S Cornelissen S Maessen JG Nia PS. Step-by-step guide for endoscopic mitral valve surgery. J Vis Surg. (2019) 5:30. 10.21037/jovs.2019.02.02

[25] Daemen JH Heuts S Olsthoorn JR Maessen JG Sardari Nia P. Mitral valve modelling and three-dimensional printing for planning and simulation of mitral valve repair. Eur J Cardiothorac Surg. (2019) 55(3):543–51. 10.1093/ejcts/ezy306 30202862

[26] Cates CU Gallagher AG. The future of simulation technologies for complex cardiovascular procedures. Eur Heart J. (2012) 33(17):2127–34. 10.1093/eurheartj/ehs155 22733836

[27] Gallagher AG O’Sullivan GC. Fundamentals of surgical simulation; principles & practices. London: Springer Verlag (2011).

[28] Nia PS Heuts S Daemen JHT Olsthoorn JR Chitwood WR Jr Maessen JG. The EACTS simulation-based training course for endoscopic mitral valve repair: an air-pilot training concept in action. Interact Cardiovasc Thorac Surg. (2020) 30(5):691–8. 10.1093/icvts/ivz323 31968097

[29] Seymour NE Gallagher AG Roman SA O’brien MK Bansal VK Andersen DK Virtual reality training improves operating room performance: results of a randomized, double-blinded study. Ann Surg. (2002) 236(4):458. 10.1097/00000658-200210000-00008 12368674 PMC1422600

